# Clinical Status and Outcome of Isolated Right Ventricular Hypoplasia: A Systematic Review and Pooled Analysis of Case Reports

**DOI:** 10.3389/fped.2022.794053

**Published:** 2022-04-21

**Authors:** Keiichi Hirono, Hideki Origasa, Kaori Tsuboi, Shinya Takarada, Masato Oguri, Mako Okabe, Nariaki Miyao, Hideyuki Nakaoka, Keijiro Ibuki, Sayaka Ozawa, Fukiko Ichida

**Affiliations:** ^1^Departments of Pediatrics, Graduate School of Medicine, University of Toyama, Toyama, Japan; ^2^Biostatistics and Clinical Epidemiology, Graduate School of Medicine, University of Toyama, Toyama, Japan; ^3^Department of Pediatrics, International University of Health and Welfare, Tokyo, Japan

**Keywords:** right ventricular hypoplasia, heart failure, cyanosis, patent foramen ovale, tricuspid valve

## Abstract

**Background:**

Isolated right ventricular hypoplasia (IRVH), not associated with severe pulmonary or tricuspid valve malformation, is a rare congenital myocardial disease. This study aims to evaluate the clinical status and outcome of IRVH.

**Methods:**

A systematic search of keywords on IRVH was conducted. Studies were searched from MEDLINE, EMBASE, Cochrane Central Register of Controlled Trials, and Igaku Chuo Zasshi (Ichushi) published between January 1950 and August 2021.

**Results:**

Thirty studies met the inclusion criteria. All of these studies were case reports and included 54 patients (25 males and 29 females). The median age of the patients was 2.5 years old (0–15.3 years). Of the 54 patients, 13 (24.1%) reported a family history of cardiomyopathy. Moreover, 50 (92.6%), 19 (35.2%), and 17 (31.5%) patients were diagnosed with cyanosis, finger clubbing, and dyspnea, respectively. Furthermore, 53 (98.2%) patients had a patent foramen ovale or an atrial septal defect (ASD). Z-score of the tricuspid valve diameter on echocardiogram was −2.16 ± 1.53, concomitant with small right ventricular end-diastolic volume. In addition, 29 (53.7%), 21 (38.9%), 7 (13.0%), and 2 (3.7%) patients underwent surgery, ASD closure, Glenn operation, and one and a half ventricular repair, respectively. Among them, nine (20.4%) patients expired, and the multivariable logistic regression analysis showed that infancy, heart failure, and higher right ventricular end-diastolic pressure were risk factors for death.

**Conclusions:**

IRVH was diagnosed early in children with cyanosis and was associated with high mortality. This systematic review and pooled analysis provided evidence to assess the of IRVH degree in order to evaluate the clinical status and outcome of IRVH.

## Introduction

Isolated right ventricular (RV) hypoplasia (IRVH), not associated with severe pulmonary or tricuspid valvar malformations, or ventricular septal defect (VSD), is a rare congenital myocardial disease. IRVH was first described in 1950 by Cooley et al. (1) and only limited case reports and case series have been published until recently (1–7). IRVH is also characterized by a small RV cavity, a patent foramen ovale (PFO) or an atrial septum defect (ASD), and a normal RV outflow tract concurrent with normally developed pulmonary valves (PV) (2). The clinical IRVH spectrum varies considerably from severe cyanosis, congestive heart failure, and early infant death to mild cyanosis (2, 8–14). This study aims to evaluate the clinical status and outcome of IRVH.

## Materials and Methods

### Eligibility

This study was conducted following the Preferred Reporting Items for Systematic Reviews and Meta-analyses guidelines (Supplementary Table 1) (15). This study protocol conformed to the ethical guidelines of the Declaration of Helsinki 1964 and was approved by the Research Ethics Committee of the University of Toyama (approval no. R2021087), Toyama, Japan.

A systematic search was conducted utilizing IRVH-related keywords, regardless of the presence or absence of clinical outcomes. IRVH was defined as (1) not having pulmonary valve stenosis or pulmonary atresia, (2) not having tricuspid valve malformation, (3) not having congenital heart disease other than PFO or ASD, or (4) not having arrhythmogenic RV cardiomyopathy or Uhl's disease. The exclusion criteria were (1) studies that did not focus on IRVH, (2) articles that did not present original research (conference abstracts, editorials, or commentaries), (3) non-human studies (animal studies or *in vitro* experiments), (4) duplicated studies, and (5) any studies that the investigators deemed irrelevant to the objective.

### Study Identification

MEDLINE, EMBASE, Cochrane Central Register of Controlled Trials on the Ovid platform, and the Japanese literature database Igaku Chuo Zasshi (Ichushi) were searched for studies published in any language between January 1950 and August 2021. The experienced librarians at the National Center for Child Health and Development, who were also affiliated with Cochrane Japan, Tokyo, Japan, performed searches using the terms described in Supplementary Table 2.

### Study Selection

Two investigators performed independent reviews of the articles. The titles and abstracts of all articles were read through during the initial screening, and articles that met the exclusion criteria were excluded. All articles were reviewed and identified for eligibility for secondary screening. A third reviewer moderated a face-to-face meeting whenever the two reviewers disagreed on an article's eligibility to determine its suitability.

### Assessment of Risk of Bias in Included Studies

The following key domains were assessed following the guidance in the Cochrane Handbook (version 5.1.0) (16): (1) random sequence generation (selection bias), (2) allocation sequence concealment (selection bias), (3) blinding of participants and personnel (performance bias), (4) blinding of outcome assessment (detection bias), (5) incomplete outcome data (attrition bias), (6) selective outcome reporting (reporting bias), and (7) other biases. Two review authors (KH and SO) independently assessed the risk of bias of included studies. Disagreements were resolved by consensus. Study authors of eligible studies were contacted to resolve uncertainties and provide further data to reduce exclusion bias and minimize missing data.

### Statistical Analysis

Continuous variables were expressed as means ± standard deviation (SD) or median [interquartile range (IQR)] values. Categorical variables were expressed as numbers and percentages. Continuous variables were compared using the unpaired *t*-test, non-parametric Mann–Whitney *U*-test, or one-way analysis of variance. However, categorical variables were compared using χ^2^ statistics or Fisher's exact test, as appropriate. Univariate regression tests were performed on all variables, and a multivariate logistic regression was performed on statistically significant variables (*P* < 0.05). The variables for inclusion were carefully selected, to ensure parsimony of the final models given the number of events. Statistical analyses were performed using the JMP software (version 13; SAS Institute, Cary, NC, USA). A *p*-value of < 0.05 was considered statistically significant.

## Results

### Literature Search and Characteristics of the Eligible Studies

**Figure 1:**
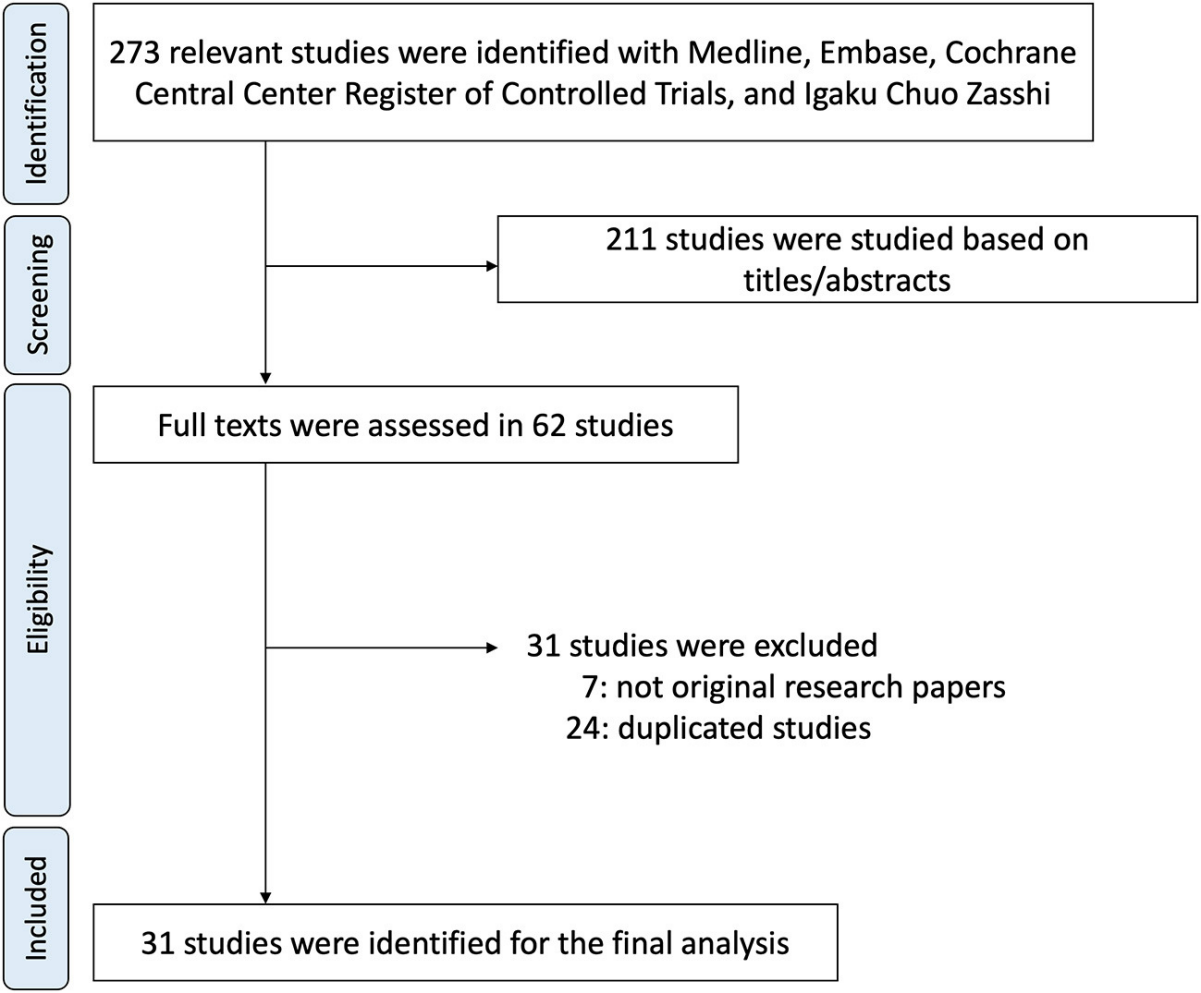
Flow chart of the study.

Four databases were utilized to identify 273 articles. Of the articles, 211 were excluded based on ineligibility determined by having titles and abstracts suggesting apparent ineligibility (Figure 1). Two investigators independently evaluated the entire contents of the remaining 62 articles and ultimately identified 31 articles as eligible for the study (2–8, 11–13, 17–38). All of these studies were case reports.

### Risk of Bias

The risk of bias was assessed for all included studies (Supplementary Table 3).

### Case Demographics

Data from 54 patients (25 males and 29 females) who had IRVH (Tables 1, 2) were obtained from the 31 included studies, all of which were case reports. The median age of the patients was 2.5 (0–15.3 years) years old. The follow-up period was 1.3 (0.3–4.3) years. Moreover, 13 (24.1%) patients reported a family history of IRVH. Furthermore, 50 (92.6%), 19 (35.2%), and 17 (31.5%) patients were diagnosed with cyanosis, finger clubbing, and dyspnea, respectively. Only three (5.6%) patients had arrhythmia.

**Table 1:** Characteristics of 54 IRVH cases from 31 studies.

**#**	**Reference**	**Age (years)**	**Sex**	**PFO/ ASD**	**FX of IRVH**	**Cyanosis**	**Finger clubbing**	**Dyspnea**	**Fatigability**	**Heart failure**	**Murmur**	**Arrhythmia**	**Angio**	**Echo**	**Surgery**	**TV**	**PV**	**Outcome**
1	Gasul et al. (17)	4	F	ASD	No	Yes	No	No	No	Yes	No	No	No	No	Yes	Normal	Normal	Alive
2	Sackner et al. (7)	0.2	M	PFO	No	Yes	No	Yes	No	Yes	No	No	No	No	No	Small	N/A	Died
3		30	M	ASD	Yes	No	No	Yes	Yes	Yes	Yes	No	Yes	No	No	Normal	N/A	Died
4		22	M	ASD	Yes	Yes	Yes	No	Yes	Yes	Yes	No	Yes	No	No	small	small	Died
5		13	F	ASD	Yes	No	No	Yes	Yes	No	Yes	No	Yes	No	No	N/A	N/A	Alive
6	Medd et al. (18)	0	M	PFO	Yes	Yes	No	No	Yes	No	No	No	Yes	No	No	Small	Normal	Died
7		0	F	PFO	Yes	Yes	No	No	No	No	No	No	Yes	No	No	Small	Normal	Died
8	Enthoven et al. (19)	23	F	PFO	No	Yes	No	No	No	No	No	No	Yes	No	Yes	N/A	N/A	Alive
9	Fay and Lynn (4)	6	F	ASD	No	Yes	Yes	No	No	No	Yes	No	Yes	No	Yes	N/A	N/A	Alive
10	Raghib et al. (20)	0	F	ASD	Yes	Yes	No	Yes	No	No	No	No	Yes	No	No	Small	Normal	Died
11	Davachi et al. (21)	0	F	ASD	Yes	Yes	No	Yes	No	No	Yes	No	Yes	No	No	Small	Normal	Died
12	Okin et al. (6)	0.3	F	ASD	No	Yes	No	No	No	No	No	No	Yes	No	No	N/A	N/A	Alive
13	Becker et al. (22)	0	M	PFO	No	Yes	No	No	No	No	Yes	No	Yes	No	Yes	N/A	Normal	Died
14	Van Der Hauwert and Michaelsson (2)	14	M	ASD	No	Yes	Yes	Yes	No	No	Yes	No	Yes	No	Yes	Normal	N/A	Alive
15		13	F	ASD	No	Yes	Yes	Yes	No	No	Yes	No	Yes	No	Yes	Normal	N/A	Alive
16	Okada et al. (32)	6	M	ASD	No	Yes	Yes	No	No	No	Yes	No	Yes	No	Yes	Normal	Normal	Alive
17	Haneda et al. (34)	6	M	ASD	No	Yes	Yes	No	No	No	Yes	No	Yes	No	Yes	Normal	Normal	Alive
18	Haneda et al. (33)	8	F	ASD	No	Yes	Yes	No	No	No	No	No	Yes	No	Yes	Normal	Small	Alive
19	Haworth et al. (23)	0.5	M	ASD	No	Yes	Yes	No	No	No	No	No	Yes	No	Yes	N/A	N/A	Alive
20		2	F	ASD	No	Yes	Yes	No	No	No	Yes	No	Yes	No	Yes	N/A	N/A	Alive
21		6	M	ASD	No	Yes	Yes	No	No	No	Yes	No	Yes	No	Yes	Small	N/A	Alive
22	Folger (25)	0	M	ASD	No	Yes	No	No	No	No	No	No	Yes	No	No	Small	Normal	Alive
23	Sugiki et al. (35)	2	F	ASD	No	Yes	Yes	No	No	No	Yes	No	No	No	Yes	Normal	Small	Alive
24	Kondo et al. (8)	0	M	ASD	No	Yes	No	No	No	No	No	No	Yes	No	No	Normal	Normal	Alive
25		0	M	ASD	No	Yes	No	No	No	No	No	No	Yes	Yes	No	Normal	Normal	Died
26	Satokawa et al. (26)	49	F	ASD	No	Yes	Yes	No	No	No	No	No	No	Yes	Yes	Normal	Normal	Alive
27	Hasegawa et al. (27)	0.06	F	ASD	No	Yes	No	No	No	No	Yes	No	Yes	No	Yes	Small	Normal	Alive
28	Wolf et al. (28)	0	F	PFO	No	Yes	No	No	No	No	Yes	Yes	Yes	Yes	Yes	Small	Normal	Alive
29	Thatai et al. (29)	1.5	M	ASD	No	Yes	Yes	No	No	No	Yes	No	Yes	Yes	Yes	Small	Small	Alive
30	Kobayashi and Arai (36)	0	M	ASD	Yes	Yes	Yes	Yes	No	No	No	No	No	Yes	No	Normal	Normal	Alive
31		0	M	PFO	Yes	Yes	No	No	No	No	No	No	No	Yes	No	Small	Normal	Alive
32	Goh et al. (5)	42	F	ASD	No	Yes	No	Yes	Yes	Yes	No	No	Yes	No	Yes	N/A	N/A	Alive
33	Ota et al. (37)	37	F	ASD	No	Yes	Yes	Yes	Yes	No	No	Yes	Yes	Yes	Yes	Normal	Normal	Alive
34	Joy et al. (30)	1	F	ASD	No	Yes	No	No	No	No	Yes	No	Yes	Yes	Yes	N/A	Normal	Alive
35		2	F	ASD	No	Yes	No	No	No	No	Yes	No	Yes	Yes	Yes	Small	Normal	Alive
36		9	M	ASD	No	Yes	No	No	No	No	Yes	No	Yes	Yes	Yes	Small	Normal	Alive
37		3	F	ASD	No	Yes	No	No	No	No	No	No	No	Yes	No	Small	Normal	Alive
38		0.1	M	ASD	No	Yes	No	No	No	No	No	No	No	Yes	No	Normal	Normal	Alive
39		1.5	F	ASD	No	Yes	No	No	No	No	Yes	No	Yes	Yes	Yes	Small	Normal	Alive
40	Chessa et al. (31)	34	M	ASD	Yes	Yes	No	No	No	No	No	No	Yes	Yes	No	Small	N/A	Alive
41		0	M	ASD	Yes	Yes	Yes	No	No	No	No	No	Yes	Yes	No	Small	Normal	Alive
42	Koriyama et al. (38)	0	M	PFO	No	Yes	No	No	No	No	No	No	No	Yes	No	Small	Small	Alive
43	Kim et al. (39)	6	F	ASD	No	Yes	No	Yes	No	No	No	No	No	Yes	Yes	Small	Normal	Alive
44	Lombardi et al. (40)	0	F	ASD	No	Yes	No	No	No	No	No	No	No	Yes	No	Normal	Normal	Alive
45		0	F	ASD	No	Yes	No	No	No	No	No	No	Yes	Yes	No	Small	Normal	Alive
46		0	M	ASD	No	Yes	No	No	No	No	No	No	No	Yes	No	Small	Normal	Alive
47	Qasim et al. (41)	9	M	No	Yes	No	No	No	No	No	No	No	No	Yes	No	Normal	Normal	Alive
48		9	F	PFO	Yes	No	No	No	No	No	No	Yes	No	Yes	No	Normal	Normal	Alive
49	Khajali et al. (42)	22	F	ASD	No	Yes	Yes	Yes	No	No	Yes	No	Yes	Yes	Yes	Normal	Normal	Alive
50		36	F	ASD	No	Yes	No	Yes	No	No	No	No	Yes	Yes	Yes	Normal	Normal	Alive
51		35	F	PFO	No	Yes	Yes	Yes	No	No	No	No	Yes	Yes	Yes	N/A	N/A	Alive
52		19	M	ASD	No	Yes	Yes	Yes	No	No	No	No	Yes	Yes	Yes	N/A	N/A	Alive
53		28	M	ASD	No	Yes	No	Yes	No	No	No	No	Yes	Yes	No	N/A	N/A	Alive
54		20	F	PFO	No	Yes	No	Yes	No	No	No	No	Yes	Yes	Yes	N/A	N/A	Alive

*PFO, patent foramen ovale; ASD, atrial septal defect; FX, family history; IRVH, isolated right ventricular hypoplasia; HF, heart failure; Angio, angiogram; Echo, echocardiogram; TV, tricuspid valve; PV, pulmonary valve; F, female; M, male; and N/A, Not applicable*.

**Table 2:** Clinical characteristics of isolated right ventricular hypoplasia from previous studies.

	**Total**	**Alive**	**Deceased**	***p*-value**
	***n* = 54**	***n* = 45**	***n* = 9**	
Age (years)	2.5 (0–15.3)	6 (0.08–16.5)	0 (0–11.1)	0.0455
Sex (male)	25 (46.3%)	19 (42.2%)	6 (66.7%)	0.2748
Family history of IRVH	13 (24.1%)	7 (15.6%)	6 (66.7%)	0.0037
Symptom				
SpO2	80.3 ± 7.1	80.5 ± 7.1	77.5 ± 10.6	0.5579
CyaNosis	50 (92.6%)	42 (93.3%)	8 (88.9%)	0.5289
Finger clubbing	19 (35.2%)	18 (40.0%)	1 (11.1%)	0.1369
Dyspnea	17 (31.5%)	13 (28.9%)	4 (44.4%)	0.4394
Fatigability	6 (11.1%)	3 (6.7%)	3 (33.3%)	0.0512
Heart failure	5 (9.3%)	2 (4.4%)	3 (33.3%)	0.0281
Arrhythmia	3 (5.6%)	17 (37.8%)	4 (44.4%)	0.723
**Modalities for diagnosis**				
Angiogram	40 (74.0%)	32 (71.1%)	8 (88.9%)	0.4182
RVEDP (mmHg)	10.6 ± 4.8	10.0 ± 4.8	14.3 ± 3.2	0.0361
Echocardiogram	28 (51.9%)	27 (60.0%)	1 (11.1%)	0.0101
TVD Z-score	−2.16 ± 1.53	−2.16 ± 1.53	N/A	
MRI	9 (16.7%)	9 (20.0%)	0 (0%)	0.3280
RVEDVI (ml/m^2^)	46.7 ± 14.4	46.7 ± 14.4	N/A	
RVEF (%)	41.9 ± 11.0	41.9 ± 11.0	N/A	
PFO/ASD	53 (98.2%)	44 (93.8%)	9 (100%)	0.1382
Surgery	29 (53.7%)	28 (62.2%)	1 (11.1%)	0.0082
ASD closure	21 (38.9%)	21 (46.7%)	0 (0%)	0.0086
SP shunt	4 (7.4%)	3 (6.7%)	1 (11.1%)	0.5289
Glenn	7 (13.0%)	7 (15.6%)	0 (0%)	0.5861
One and a half repair	2 (3.7%)	2 (4.4%)	0 (0%)	1.0000
Fontan	1 (1.9%)	1 (2.2%)	0 (0%)	1.0000
Death	9 (16.7%)	0 (0%)	9 (100%)	<0.0001

*IRVH, isolated right ventricular hypoplasia; SpO2, arterial oxygen saturation; RVEDP, right ventricular end-diastolic pressure on angiogram; TVD Z-score, Z-score of the diameter of the tricuspid valve on echocardiogram; RVEDVI, index of right ventricular end-diastolic volume on cardiac magnetic resonance imaging; RVEF, right ventricular ejection fraction on angiogram; PFO, patent foramen ovale; ASD, atrial septal defect; SP, shunt systemic-to-pulmonary artery shunt; Glenn, Glenn operation; and Fontan, Fontan surgery*.

### Cardiovascular Characteristics

Of the patients, 40 (74.0%), 28 (51.9%), and nine (16.7%) were diagnosed by angiogram, echocardiogram, and magnetic resonance imaging (MRI), respectively. In addition, 11 (20.4%) and 42 (77.8%) patients had PFO and ASD, respectively. The Z-score of the diameter of the tricuspid valve on echocardiogram was−2.16 ± 1.53, concomitant with small right ventricular end-diastolic volume (RVEDV; Tables 2, 3). The right ventricular end-diastolic pressure (RVEDP) was lower in the survivors than that in the deceased (*p* = 0.0361).

**Table 3:** Summary of surgical and medical management of previous studies on patients with isolated right ventricular hypoplasia (from 2000 to 2021).

**#**	**Reference**	**Age (years)**	**Sex**	**PFO/ASD**	**Symptoms**	**SpO2 (%)**	**TVD Z-score**	**RVEDVI (ml/m^**2**^)**	**RVEF (%)**	**RVEDP (mmHg)**	**Type of surgery**	**Medical treatment**	**Outcome**
40	Chessa et al. (31)	34	M	ASD	Cyanosis	N/A	N/A	N/A	N/A	N/A	None	None	Alive
41		0	M	ASD	Cyanosis, finger clubbing	N/A	N/A	N/A	N/A	N/A	None	None	Alive
42	Koriyama et al. (38)	0	M	PFO	Cyanosis	70	−4.21	N/A	64	N/A	None	O_2_, NO, NTG	Alive
43	Kim et al. (39)	6	F	ASD	Cyanosis, dyspnea	76	−3.5	79	N/A	N/A	ASD closure, then one and half ventricle repair and tricuspid annuloplasty	Diuretics	Alive
44	Lombardi et al. (40)	0	F	ASD	Cyanosis	70	−1.29	N/A	N/A	N/A	None	O_2_	Alive
45		0	F	ASD	Cyanosis	88	−2.9	N/A	N/A	N/A	None	Dobutamine, mechanical ventilation	Alive
46		0	M	ASD	Cyanosis	86	−1.1	N/A	N/A	N/A	None	O_2_	Alive
47	Qasim et al. (41)	9	M	No	No	N/A	0.77	N/A	N/A	N/A	None	None	Alive
48		9	F	PFO	arrhythmia	N/A	0.04	N/A	N/A	N/A	None	None	Alive
49	Khajali et al. (42)	22	F	ASD	Cyanosis, finger clubbing, dyspnea, murmur	85	N/A	38	45	17	ASD closure	Diuretics	Alive
50		36	F	ASD	Cyanosis, dyspnea	86	N/A	45	35	18	ASD closure, then one and half ventricle repair	None	Alive
51		35	F	PFO	Cyanosis, finger clubbing, dyspnea	78	N/A	41	36	20	Glenn	None	Alive
52		19	M	ASD	Cyanosis, finger clubbing, dyspnea	82	N/A	39	30	12	Glenn and semiclosure of ASD	None	Alive
53		28	M	ASD	Cyanosis, dyspnea	79	N/A	40	42	11	One and half ventricle repair was refused	Diuretics	Alive
54		20	F	PFO	Cyanosis, dyspnea	88	N/A	45	41	10	ASD closure	Diuretics	Alive

*PFO, patent foramen ovale; ASD, atrial septal defect; SpO2, arterial oxygen saturation; TVD Z-score, Z-score of the diameter of the tricuspid valve on echocardiogram; RVEDVI, index of the right ventricular end-diastolic volume on cardiac magnetic resonance imaging; RVEF, right ventricular ejection fraction on angiogram; RVEDP, right ventricular end-diastolic pressure on angiogram; Glen, Glenn operation; O_2_, Oxygen; NO, nitric oxide; NTG, nitroglycerin; and N/A, Not applicable*.

### Surgical Treatment

**Figure 2:**
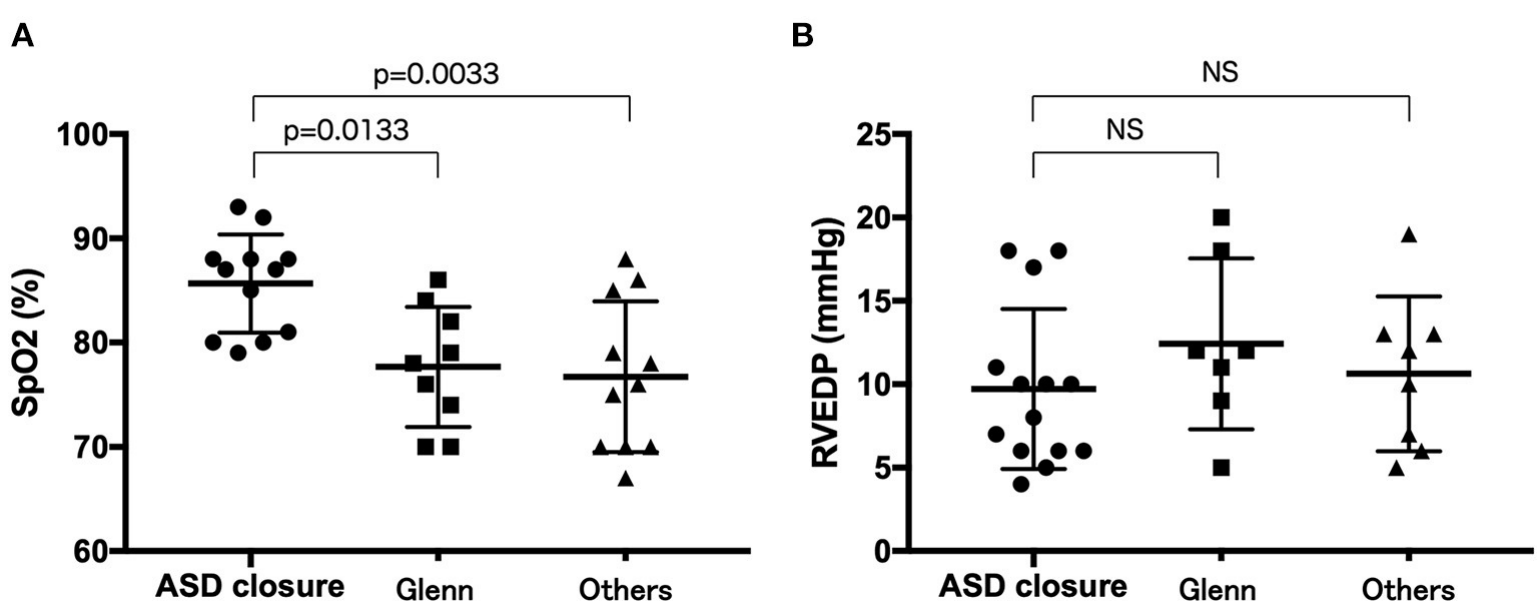
Comparison of arterial oxygen saturation **(A)** and right ventricular end-diastolic pressure **(B)** between the patients who underwent ASD closure, Glenn operation, or one and a half ventricular repair and those who did not underwent surgery. *ASD closure* patients who underwent ASD closure, *Glenn* patients who underwent Glenn operation or one and a half ventricular repair, *others* patients who did not undergo surgery.

A total of 29 (53.7%) patients underwent surgery. Specifically, 21 (38.9%), 4 (7.4%), 7 (13.0%), 2 (3.7%), and 1 (5.9%) patient underwent ASD closure, systemic-to-pulmonary artery (SP) shunt placement, a Glenn operation, one and a half ventricular repair, and Fontan surgery (Tables 2, 3). Moreover, four patients underwent multiple surgeries; two patients had SP shunt twice and the other two patients had ASD closure and then one and a half ventricular repair. Arterial oxygen saturation was higher in patients who underwent ASD closure than that in patients who underwent Glenn operation or one and a half ventricular repair or the patient without surgery (Figure 2).

### Clinical Outcomes

Nine (16.7%) patients expired; seven and two patients were diagnosed at <1 year and in adulthood, respectively. The multivariable logistic regression analysis showed that <1 year at diagnosis, heart failure, and >12 mmHg of RVEDP were independent risk factors for death (*p* < 0.05, respectively; Table 4).

**Table 4:** Univariable and multivariable logistic regression analyses for independent predictors of mortality.

	**Univariate**			**Multivariate**		
	**Odds ratio**	**95% CI**	***p*-value**	**Odds ratio**	**95% CI**	***p*-value**
Male	2.737	0.6373–14.287	0.1778			
Age <1 y	7.750	1.633–56.660	0.0089	9.962 ×10^7^	2.194 –	0.0168
Family history of IRVH	10.857	2.325–62.250	0.0024			
Heart failure	10.75	1.504–95.885	0.0191	6.395 ×10^14^	9.273 –	0.0027
RVEDP > 12 mmHg	8.003 ×10^7^	3.6275–	0.0029	8.596 ×10^13^	5.771 –	0.0027

*CI, confidence interval; IRVH, isolated right ventricular hypoplasia; RVEDP, right ventricular end-diastolic pressure on angiogram*.

## Discussion

This study revealed that IRVH was diagnosed early in children with cyanosis and was associated with high mortality between 1959 and 2000. It is believed that this is the first systematic review to evaluate the association between IRVH and surgery.

IRVH is characterized by trabecular musculature underdevelopment and a small RV cavity (2). An ASD or a PFO serves as an escape route causing a right-to-left shunt, resulting in cyanosis. This clinical symptom has a wide outcome spectrum, from mortality in early infancy to mild cyanosis. Congestive heart failure and deep cyanosis can appear during infancy in seven cases, whereas symptoms (e.g., mild cyanosis, dyspnea, and clubbed finger) may be found later in cases with less malformation. This may depend on hypoplasia degree and interatrial communication size.

From the systematic review and pooled analysis of the current study, IRVH was frequently observed in those patients diagnosed at <1 year. Thus, IRVH may be thought of as a primary developmental RV anomaly or that it may be due to a reduced tricuspid flow during the fetal stage (18). The resultant restrictive physiology reduces RV inflow *via* a detrimental feedback loop when the tricuspid size and RV compliance are decreased because of RV hypoplasia. In addition, the blood entering the RV is reduced further as the inflow of blood flowing from the right to the left atrium *via* a PFO or an ASD is increased.

Diagnostic tests are crucial to the differential diagnosis of IRVH from other diseases with cyanosis, and the diagnosis and evaluation of the syndrome severity were routinely performed by angiogram, echocardiography, and MRI. Cardiac catheterization can provide precise hemodynamics including RV filling and capacity quantification of the right-to-left shunts *via* ASD or PFO. MRI can provide comprehensive evaluations, and is widely used in the assessment of congenital heart defects. Moreover, MRI provides precise data on ventricular function and volume (43). Although the change of modalities may reflect the historical transition from angiography to echocardiography or MRI, combining multimodalities to assess IRVH hemodynamics and morphology is crucial to diagnose and make therapeutic management.

The surgical option may also vary according to the severity of RV hypoplasia from biventricular repair with ASD closure to univentricular repair with a Glenn operation and one and a half ventricular repair (2, 11, 23, 30, 33, 44). Although cyanosis was found in both the living and deceased patients, heart failure, reflecting on higher RVEDP, was observed more frequently in deceased patients, indicating that ASD closure could be undertaken in patients with mild IRVH. These data suggested that surgical options were limited for deceased patients in whom IRVH was more severe and symptomatic. Thus, right-to-left atrial communication is unnecessary for survival if RV hypoplasia is less severe, and such patients are expected to have a good prognosis. In contrast, the right-to-left atrial communication is necessary for survival if RV hypoplasia is more severe, and such patients are expected to have cyanosis and a poor prognosis, indicating Glenn operation or one and a half ventricular surgery. Thus, arterial oxygen saturation may be a predictive maker for deciding on surgery to assess the cyanosis degree.

Extrapolating from certain congenital heart anomalies with diastolic dysfunction of the right ventricle, such as severe pulmonary stenosis or atresia, certain mild to moderate forms of Ebstein's disease, in which the question arises of the feasibility of closure of the interatrial communication, in this type of pathophysiology of right to left interatrial shunt, a balloon occlusion test would be necessary before closing this communication. Percutaneous closure will follow in favorable cases.

Interestingly, the results of the current study showed that 24.1% of the patients had a family history of IRVH. Several reports have suggested that the mode of IRVH inheritance is an autosomal-dominant pattern (7, 18, 20, 21, 31, 36). Although genetic variants have not been reported, higher IRVH inheritance and positive family history is a risk factor for survival which may indicate that IRVH may be caused by a genetic disorder.

### Study Limitations

Quantitative analyses were not performed in this systematic review due to the heterogeneity between studies and the limited amount of data in addition to the IRVH ambiguous definition. Any randomized controlled trials were not included because all the included studies were cohort studies and case series with small sample sizes. Therefore, we conducted a pooled analysis of these case report data to analyze factors related to life expectancy. Numerical data including RV size, tricuspid valve diameter, pulmonary valve diameter, or ASD size, could not be obtained from all the included studies. Therefore, the classification of cardiac phenotypes was subjective. This study considered >70 years, Treatments were developed that improved disease outcomes during the study period, which may have altered the results of the study. Several of the studies were retrospective and thus did no not perform long-term patient follow-up.

## Conclusion

This systematic review and pooled analysis provided evidence to assess IRVH degree, and evaluate the clinical status and outcome of IRVH. Combining multiple modalities (e.g., cardiac MRI, echocardiography, and angiography) may be important in the diagnosis and treatment of each patient because the IRVH severity varies. However, the IRVH etiology has not yet been elucidated. The registry study we are conducting is ongoing. Further studies are warranted to reveal the IRVH etiology, including its genetic background and hemodynamic evaluation, which will lead to better IRVH management and treatment.

## Data Availability Statement

The original contributions presented in the study are included in the article/Supplementary Material, further inquiries can be directed to the corresponding author.

## Author Contributions

All authors listed have made a substantial, direct, and intellectual contribution to the work and approved it for publication.

## Funding

KH was supported by grants from The Ministry of Education, Culture, Sports, Science and Technology in Japan (Grant-in-Aid for Scientific Research Nos. 18K07785 and 21K08124).

## Conflict of Interest

The authors declare that the research was conducted in the absence of any commercial or financial relationships that could be construed as a potential conflict of interest.

## Publisher's Note

All claims expressed in this article are solely those of the authors and do not necessarily represent those of their affiliated organizations, or those of the publisher, the editors and the reviewers. Any product that may be evaluated in this article, or claim that may be made by its manufacturer, is not guaranteed or endorsed by the publisher.

## Abbreviations

IRVH: Isolated right ventricular hypoplasia
HF: Heart failure
RV: Right ventricular
PV: Pulmonary valves
PFO: Patent foramen ovale
ASD: Atrium septum defect
TV: Tricuspid valve
RVEDV: Right ventricular end-diastolic volume
RVEDP: Right ventricular end-diastolic pressure.
